# Supplementary material for: Using Mindful Attention Awareness Scale on male prisoners: Confirmatory factor analysis and Rasch models
Source: PLoS One. 2021 Jul 13;16(7):e0254333. doi: 10.1371/journal.pone.0254333 (PMC8277060; doi:10.1371/journal.pone.0254333)
Supplement: Attachment — Submitted filename: 200418_Response letter.doc [file pone.0254333.s002.doc]

**Dear Editor-in-Chief,**

Thank you for inviting us to revise our manuscript entitled “Using Mindful Attention Awareness Scale on male prisoners: confirmatory factor analysis and Rasch models” (PONE-D-20-09274).

Below we have provided our point-by-point reply to the comments made by the previous academic editor.

We look forward to your further comments.

Sincerely yours,

Amir H. Pakpour, PhD

Chung-Ying Lin, PhD

Response to Comments:

**1. The methods are unclear. Did you use CFA for ordinal indicators (items) as implemented in Mplus or the R-package lavaan ?**

*Reply*: We have now provided the software information in the revised.

“Apart from the descriptive statistics (mean and SD for continuous variables; frequency and percentage for categorical variables), confirmatory factor analysis (CFA) using AMOS 24.0 and Rasch analysis using WINSTEPS 4.3.0 (winsteps.com) were used to test the construct validity of the Persian MAAS among male prisoners.”

**2. The results are lacking. You should report chi-square, df and P-value for the CFA fit. You should also report the RMSEA and corresponding confidence interval. I would be appropriate to cite literature indicating the type I error rates associated with the Rasch item fit statistics. You do not report on local response dependence - this is unacceptable.**

*Reply*: We have now clearly stated the information.

“The one-factor structure of the MAAS is verified by the fit indices of the CFA, including CFI (0.928), TLI (0.908), RMSEA (0.063), 95% CI of RMSEA (0.052, 0.074), and SRMR (0.049), except for the significant χ^2^ test (Table 2).”

“Rasch analysis further shows that The global test on type I error rates associated with the Rasch item fit statistics was nonsignificant (p = 0.48), indicating the satisfactory item fit across all the items.”

“Tests on local response dependence indicate that there were no substantial residual correlations among the items (r = -0.33 to 0.27).”

**3. How can you state that 'CFA results showed a single factor solution for the Persian MAAS', when CFI=0.928 and TLI=0.908 ?**

*Reply*: The practice of CFI and TLI > 0.9 to indicate data-model fit is widely used in the literature, including those published recently. Also, statistics textbook also recommends the cutoff at 0.9. Therefore, we are confident that using CFI = 0.928 and TLI = 0.908 (together with other CFA fit indices) can support the decision of a single factor solution for the Persian MAAS.

*Ref:*

Hoyle, R. H., & Panter, A. T. (1995). Writing about structural equation modeling. In R. H. Hoyle (Ed.), Structural equation modeling: Concepts, issues, and application. Thousand Oaks, CA: Sage.

Kline, R. B. (1998). Principles and practice of structural equation modeling. New York: Guilford Press.

Leung, H., Pakpour, A. H., Strong, C., Lin, Y.-C., Tsai, M.-C., Griffiths, M. D., Lin, C.-Y., Chen, I.-H. (2020). Measurement invariance across young adults from Hong Kong and Taiwan among three internet-related addiction scales: Bergen Social Media Addiction Scale (BSMAS), Smartphone Application-Based Addiction Scale (SABAS), and Internet Gaming Disorder Scale-Short Form (IGDS-SF9)(Study Part A). Addictive Behaviors, 101, 105969.

Chen, I.-H., Strong, C., Lin, Y.-C., Tsai, M.-C., Leung, H., Lin, C.-Y., Pakpour, A. H., Griffiths, M. D. (2020). Time invariance of three ultra-brief internet-related instruments: Smartphone Application-Based Addiction Scale (SABAS), Bergen Social Media Addiction Scale (BSMAS), and the nine-item Internet Gaming Disorder Scale- Short Form (IGDS-SF9) (Study Part B). Addictive Behaviors, 101, 105960.

Pakpour, A. H., Tsai, M.-C., Lin, Y.-C., Strong, C., Latner, J. D., Fung, X. C. C., Lin, C.-Y., & Tsang, H. W. H. (2019). Psychometric properties and measurement invariance of the Weight Self-Stigma Questionnaire and Weight Bias Internalization Scale in Hongkongese children and adolescents. International Journal of Clinical and Health Psychology, 19, 150-159.
