# Supplementary material for: Using Mindful Attention Awareness Scale on male prisoners: Confirmatory factor analysis and Rasch models
Source: PLoS One. 2021 Jul 13;16(7):e0254333. doi: 10.1371/journal.pone.0254333 (PMC8277060; doi:10.1371/journal.pone.0254333)
Supplement: Attachment — Submitted filename: 210206_Response letter.doc [file pone.0254333.s003.doc]

**Dear Dr. Vousden,**

Thank you for inviting us to revise our manuscript entitled “Using Mindful Attention Awareness Scale on male prisoners: confirmatory factor analysis and Rasch models” (PONE-D-20-09274R1).

Below we have provided our point-by-point reply to the comments made by the three reviewers. All the revisions are presented using red fonts in the revised manuscript. We deeply appreciate their comments, which help us substantially improve our work.

We look forward to your further comments.

Sincerely yours,

Amir H. Pakpour, PhD

Chung-Ying Lin, PhD

Response to Editor:

**1. Please note that as per our publication criteria, PLOS ONE requires that all experiments, statistics and other analyses are performed to a high technical standard, described in sufficient detail and adhere to appropriate reporting guidelines and community standards. Conclusions must be presented in an appropriate fashion and be supported by the data (Please see http://journals.plos.org/plosone/s/criteria-for-publication). The reviewers’ comments concern the statistical analysis performed, including the justification for the methods employed and the ability of the results to support the conclusions.**

*Reply*: We have now clearly responded to the reviewers’ comments regarding the justifications of choosing specific statistical methods. Please see our detailed response to Reviewer #1’s comment 4.

**2. One of the reviewers has also provided comments encouraging you to engage in open science practices. All PLOS journals require authors to make all data necessary to replicate their study’s findings publicly available without restriction at the time of publication. When specific legal or ethical restrictions prohibit public sharing of a data set, authors must indicate how others may obtain access to the data. Authors must share the “minimal data set” for their submission. PLOS defines the minimal data set to consist of the data required to replicate all study findings reported in the article, as well as related metadata and methods. (Please see here: https://journals.plos.org/plosone/s/data-availability).**

**I note that you have indicated that all data are fully available without restriction all relevant data are within the manuscript and its Supporting Information files. However, no Supporting Information files have been included. In your revised manuscript please ensure that you provide details of where your data are deposited and how these can be accessed. More information on data deposition methods is provided in the link provided in the previous paragraph. Whilst only data sharing is a requirement for publication, I do encourage to make your scripts available to further improve the reproducibility of your work. If you provide any materials used in your study as Supporting Information files please ensure that you have permission to reproduce these with a CC BY license (please see here for more details: https://journals.plos.org/plosone/s/licenses-and-copyright).**

*Reply*: We have now added the dataset but we believe that our data is partly sensitive because they are prisoners. We need to make sure from our institution if we can publish our whole dataset.

**3. Please describe what considerations were made for the prisoners included in this study. For instance, were participants able to opt out of the study? Did individuals who did not participate receive the same treatment offered to participants?**

*Reply*: Thanks for your comment. The participation to the study was voluntary and anonymous. There were no any interventional materials (or treatment) as this study was a cross-sectional study. The participants were only asked to complete the study measure after describing study aims and checking eligibility criteria.

Response to Reviewer #1:

**1. After reviewing the new manuscript and the responses to the reviewers, I consider that this version meets all the requirements to be published in Plos One as an original article. I have no further comment to make.**

*Reply*: Thank you for the positive comment.

Response to Reviewer #2:

**1. Overall, I see added value on this paper. However, there are several necessary changes (especially regarding the introduction and theoretical sections) that need to occur prior to acceptance. I provide a list below. Perhaps the best added value of this contribution is the access to a big hard-to-reach sample of prisoners from a non-WEIRD population. A focus on this would improve the paper significantly.**

*Reply*: Thank you for the positive comment. We also appreciate your following comments, which help us substantially improve this work.

**2. Abstract:**

**-Fit indices are not required in the abstract, consider removing them.**

*Reply*: We have now removed the fit indices from the Abstract.

“The CFA results showed a single factor solution for the Persian MAAS.”

**3. Introduction:**

**-The whole introduction needs restructuring. While I see added value on validating the MAAS in this population and I see worthy work in the authors' efforts, the core messages of the introduction are misguided. The MAAS is right now an outdated instrument for Western scenarios (e.g. integration in other instruments, and content validity issues since it measures Mindlessness or Automatic Pilot, not mindfulness), used in laboratory settings due to its simplicity. However, it is important to provide validated instruments world-wide, so I see value in this paper. A restructured introduction in this direction would be a great (and necessary) plus for the paper (and accordingly, changes in discussion). Some other inquiries are provided below.**

**-MBIs count with heavier evidence than those provided. Consider adding reviews or meta-analyses when picturing evidence on MBIs (e.g. Creswell, 2017, Annual review of Psychology).**

**-Current evidence on mindfulness models rely strongly on multidimensional definitions (e.g. Lindsay & Creswell, 2017; Hölzel et al, 2011).**

**-Psychometric developments on mindfulness state clearly that the MAAS has been already integrated in other multidimensional instruments (e.g. FFMQ).**

*Reply*: Thank you for the guidance in the Introduction and also the valuable references. We have now restructured the Introduction with the incorporation of your suggested references.

“Moreover, a review summarized the effectiveness of MBIs through evaluating randomized controlled trials (RCTs) and found that the analyzed rigorous RCTs showing the effectiveness of MBIs on different outcomes, including addition, chronic pain, and depression relapse [12].”

“The concept of “mindfulness” has been argued for its operational definition [13] because of the arguments on whether mindfulness is a single factor [1,14,15] or a multifactor [5,16] construct. However, current evidence on mindfulness models is prone to multifactor [17,18].”

“Although the MAAS suffers from some problems in its content validity (i.e., only assessing part of the mindfulness via mindlessness facet) and becomes outdated in the Western country, it deserves a breaking point for the countries without appropriate instruments assessing mindfulness.”

“In addition, the MAAS has been integrated into other mindfulness instruments to assess the nature of multifactor structure in mindfulness (e.g., the Five Facet Mindfulness Questionnaire) [26].”

*References:*

12.	Creswell JD. Mindfulness interventions. Annu Rev Psychol. 2017;68(1):491-516.

17.	Hölzel BK, Carmody J, Vangel M, et al. Mindfulness practice leads to increases in regional brain gray matter density. Psychiatry Res. 2011;191(1):36-43. doi:10.1016/j.pscychresns.2010.08.006

18.	Lindsay EK, Creswell JD. Mechanisms of mindfulness training: Monitor and Acceptance Theory (MAT). Clin Psychol Rev. 2017;51:48-59. doi:10.1016/j.cpr.2016.10.011

26.	Baer RA, Smith GT, Hopkins J, Krietemeyer J, Toney L. Using self-report assessment methods to explore facets of mindfulness. Assessment. 2006;13(1):27-45. doi:10.1177/1073191105283504

**4. Methods:**

**- I see of great value to provide such a big sample for a hard-to-reach scenario such as prisons. My congratulations.**

**- Instruments are provided with Cronbach's alpha. Although this is widely used, is flawed (see McNeish, 2018, Viladrich, Angulo-Brunet & Doval, 2017, and Trizano-Hermosilla & Alvarado, 2016, for references). Given all self-reports are ordinal and probably with normality or tau-equivalence issues, I recommend to remove alpha and provide mcdonald's omega, guttman's lambda6, GLB or other similar indices. The JASP software is a free and effective tool to obtain these indices (download at: https://jasp-stats.org/download/)**

**- CFA was used with maximum likelihood when ordinal items were implemented. This is open to method bias since ML assumes items to be continuous and normal. Current estimation methods for ordinal items are (1) Weighted Least Squares Mean-and-Variance Adjusted (WLSMV) or Maximum Likelihood Robust (MLR), and (2) polychoric correlations. If the authors encounter software limitations, MPlus and lavaan package of R provide all these requirements.**

**- Why Rasch models? There is no justification on why these models are better than other IRT models (e.g. Likelihood Ratio Tests for 1 (Rasch), 2, 3 or 4 paremeter logistics models). Finally, why rating scale model? Why not, for example, the graded response model? These choices need to be justified. In addition, local independence check methods need to be specified in the Data Analysis section.**

**- It would be interesting to test external validity of the MAAS in this sample beyond DIF. For example, correlation analysis for the MAAS scores with the ISI and GHQ scores.**

**- Psychology is right now facing a reproducibility crisis (see Munafó et al, 2017, for details). Is there a possibility for the authors engaging in open science practices? For example, sharing openly in the Open Science Framework the data, scipts, and other materials to ensure reproduction of results. Since the data may encounter privacy issues due to prison environment, this is not a request but a suggestion for general improvement of psychological science. In any case, I did not see any statement on this matter in the manuscript.**

*Reply*: Thank you. We have now made the following revisions in the Methods and Results.

(1) Both Cronbach’s alpha and McDonald’s omega are presented for the used instruments.

“Moreover, the internal consistency of the MAAS was adequate (Cronbach’s α=0.878; McDonalds’ ω=0.879).”

“Moreover, the internal consistency of the ISI was adequate (Cronbach’s α=0.900; McDonalds’ ω=0.902).”

“Moreover, the internal consistency of the GHQ-12 was adequate (Cronbach’s α=0.714; McDonalds’ ω=0.653).”

(2) We have now used weighted least squares mean-and-variance adjusted (WLSMV) estimator in the CFA. The results are revised accordingly (Please refer to Tables 2 and 3).

“In the CFA, a one-factor structure was tested for the MAAS using the diagonally weighted least squares (DWLS) estimator.”

“The one-factor structure of the MAAS is supported by the fit indices of the CFA, including CFI (0.982), TLI (0.979), RMSEA (0.044), 95% CI of RMSEA (0.032, 0.055), and SRMR (0.064), except for the significant χ^2^ test (Table 2). Moreover, all the factor loadings in the MAAS were strong (0.478 to 0.679) and significant (Table 3).”

(3) We have now provided the justifications regarding the use of Rasch model and rating scale model. The local independence check methods are clearly mentioned in the Data Analysis section.

“We considered using Rasch model with RSM instead of other item-response theory models (e.g., the two parameter or the three logistic parameter model with partial credit model or graded response model) because Rasch model with RSM can provide simpler estimation in modeling (i.e., Rasch model with RSM needs not to estimate other parameter like discrimination and the category difference for every two responses). Thus, the benefit of using such a model than other types of item-response theory model is it fits better with the principle of parsimony.”

“Local independence was tested to understand whether residual correlations exist among items. Specifically, we tested the Rasch residual for every item and used the correlations between the Rasch residuals to examine the local independence, where an absolute correlation coefficient > 0.4 indicating substantial dependence.”

(4) The correlations between the MAAS, ISI, and GHQ scores are conducted and presented.

“Apart from the CFA and Rasch, Pearson correlations were carried to understand the associations between the MAAS, the ISI, and the GHQ-12 scores.”

“Moreover, the MAAS total score was significantly correlated with the ISI (r=-0.60; p<0.001) and the GHQ-12 (r=-0.44; p<0.001) scores.”

(5) We have now added the dataset but we believe that our data is partly sensitive because they are prisoners. We need to make sure from our institution if we can publish our whole dataset.

**5. Discussion:**

**- "Our Rasch analysis results corresponded to our CFA results; that is, both psychometric testing supported the one-factor structure of the MAAS and no items were misfit". This is relatively obvious since the same sample was applied. Consider removing.**

**- The practical implications need to consider the content validity issues of the MAAS (measuring the mindlessness facet of mindfulness).**

*Reply*: We have now removed the sentence “Our Rasch analysis results corresponded to our CFA results; that is, both psychometric testing supported the one-factor structure of the MAAS and no items were misfit”. Also, we have mentioned the content validity issues of the MAAS in the practical implications.

“However, cautious should be paid attention to because the content validity of the MAAS only contributes to the mindlessness facet of mindfulness. Therefore, healthcare providers may want to integrate the MAAS with other mindfulness instrument to evaluate the effects of mindful programs.”

Response to Reviewer #3:

**1. Page 3: Reference to mediation in second paragraph. I'm not clear on why the general population's experience of meditation relates to the factor structure of mindfulness as a construct.**

*Reply*: We have now revised the descriptions here.

“Nevertheless, a single factor (i.e., attention and awareness of the present) can be used as the basis to assess mindfulness before expanding the concept of mindfulness to pose various dimensionalities, considering that a single-factor structure is easier to be studied and understood than a multi-factor structure.”

**2. Page 4, first paragraph: "brief state" versions - do you mean brief versions, or versions of the MAAS intended to measure state vs trait mindfulness?**

*Reply*: They are brief versions. We have now corrected the descriptions.

“Additionally, several brief versions of the MAAS have been developed and validated (e.g., [21,22])”

**3. Results:**

**The term "verified" is used to describe a one-factor structure, this wording is too strong. First, though many of the fit statistics are acceptable, the RMSEA is outside of the good fit range, and these statistics are often most useful in comparison to other models (i.e., there could be a 3 factors structure with even better fit statistics). Are there are factor structures of the MAAS that have been proposed or found (such as the Iranian study cited in the Discussion)? If so, please test in comparison. If not, please just change wording to "one-factor structure is supported by the fit indices"**

*Reply*: Thank you for the guidance. We have now revised the wording to “supported”.

“The one-factor structure of the MAAS is supported by the fit indices of the CFA, including CFI (0.928), TLI (0.908), RMSEA (0.063), 95% CI of RMSEA (0.052, 0.074), and SRMR (0.049), except for the significant χ^2^ test (Table 2)”

**4. Also, I think it would be valuable for this study to report descriptive statistics of the MAAS and compare the values in this population to others in other samples.**

*Reply*: We have now described the descriptive statistics and compared with other studies.

“On average, the MAAS mean score was 4.21 (0.91).”

“Moreover, the MAAS mean score found in the present sample of prisoners was slightly higher than that of college students (MAAS mean score=3.89 for Bangla students [19]; 3.88 for Greek students [44]; and 3.72 to 4.01 for American students [1,30,45]) but lower than that of general population (MAAS mean score=4.86 for Italians [23] and 4.45 for Canadians [46]).”
