# Supplementary material for: Using Mindful Attention Awareness Scale on male prisoners: Confirmatory factor analysis and Rasch models
Source: PLoS One. 2021 Jul 13;16(7):e0254333. doi: 10.1371/journal.pone.0254333 (PMC8277060; doi:10.1371/journal.pone.0254333)
Supplement: Attachment — Submitted filename: 210206_Response letter.doc [file pone.0254333.s004.doc]

**Dear Dr. Vousden,**

Thank you for inviting us to revise our manuscript entitled “Using Mindful Attention Awareness Scale on male prisoners: confirmatory factor analysis and Rasch models” (PONE-D-20-09274R2).

Below we have provided our point-by-point reply to the comments made by the three reviewers. All the revisions are presented using red fonts in the revised manuscript. We deeply appreciate their comments, which help us substantially improve our work.

We look forward to your further comments.

Sincerely yours,

Amir H. Pakpour, PhD

Chung-Ying Lin, PhD

Response to Reviewer #2:

**1. - There is no justification on why estimating correlations between instrument scores with Pearson correlations. Assumption checks for normality and outliers is necessary to ensure Pearson's r estimates the correlations adequately, and if not, use alternative estimators (e.g. Spearman's rho or Kendall's tau-b).**

*Reply*: We have conducted Spearman's rho correlations for the above mentioned associations.

**2. - "Western country" instead of "Western countries" (p. 5).**

**- "Modern test theory" instead of "item response theory" (p. 6).**

*Reply*: We have now edited these. Thanks
