# Supplementary material for: Using Mindful Attention Awareness Scale on male prisoners: Confirmatory factor analysis and Rasch models
Source: PLoS One. 2021 Jul 13;16(7):e0254333. doi: 10.1371/journal.pone.0254333 (PMC8277060; doi:10.1371/journal.pone.0254333)
Supplement: Attachment — Submitted filename: 210602_Response letter.doc [file pone.0254333.s005.doc]

**Dear Prof. Dr. van Luijtelaar,**

Thank you for inviting us to revise our manuscript entitled “Using Mindful Attention Awareness Scale on male prisoners: confirmatory factor analysis and Rasch models” (PONE-D-20-09274R3).

Below we have provided our point-by-point reply to the comments made by the three reviewers. All the revisions are presented using red fonts in the revised manuscript. We deeply appreciate their comments, which help us substantially improve our work.

We look forward to your further comments.

Sincerely yours,

Amir H. Pakpour, PhD

Chung-Ying Lin, PhD

Response to editor:

**1. - Please add a rationale, after checking for outliers and normality of the distributions, for using a parametric correlation coefficient, or use a non-parametric one to be on the safe side.**

**The reviewer was happy with all the changes made by you. A minor issue that the reviewer noticed was that there was no justification on why estimating correlations between instrument scores with Pearson correlations. Assumption checks for normality and outliers are necessary to ensure Pearson's r estimates the correlations adequately, and if not, use alternative estimators (e.g. Spearman's rho or Kendall's tau-b).**

*Reply*: Thank you for the positive feedback. We agree that using nonparametric estimator for the correlations is better. Therefore, we have now conducted Spearman's rho correlations for the above mentioned associations.

“Apart from the CFA and Rasch, Spearman’s rho correlations were carried to understand the associations between the MAAS, the ISI, and the GHQ-12 scores.”

“Moreover, the MAAS total score was significantly correlated with the ISI (r=-0.601; p<0.001) and the GHQ-12 (r=-0.384; p<0.001) scores.”

**2. - "Western country" instead of "Western countries" (p. 5).**

**- "Modern test theory" instead of "item response theory" (p. 6).**

*Reply*: We have now edited these. Thanks

“and becomes outdated in the Western countries,”

“Then, Rasch analysis from the item response theory was applied to evaluate”
